# Lower risk of liver cancer in patients with schizophrenia: a systematic review and meta-analysis of cohort studies

**DOI:** 10.18632/oncotarget.21679

**Published:** 2017-10-09

**Authors:** Dali Xu, Guangdong Chen, Lingguang Kong, Wei Zhang, Lirong Hu, Ce Chen, Jie Li, Chuanjun Zhuo

**Affiliations:** ^1^ Department of Psychiatry, Wenzhou Seventh People's Hospital, Wenzhou, 325000, China; ^2^ Department of Psychiatry, Tianjin Anding Hospital, Tianjin Mental Health Center, Tianjin, 300222, China

**Keywords:** schizophrenia, liver cancer, incidence, cohort study, systematic review

## Abstract

Previous studies regarding the association between schizophrenia and the subsequent risk of liver cancer have shown inconsistent results. We aimed to perform a systematic review and meta-analysis to evaluate the association between schizophrenia and liver cancer incidence. We systematically searched the PubMed and Embase electronic databases for cohort studies reporting the standardized incidence ratio (SIR) for the risk of liver cancer in patents with schizophrenia as compared with the general population. A random-effects model was used to analyze the data. Stratified analyses were performed according to the gender of the patients. Seven studies comprising 312,834 patients with schizophrenia were included. During follow-up, 581 liver cancer cases were confirmed. The meta-analysis results showed that schizophrenia was associated with a trend of a lower liver cancer incidence (SIR: 0.83, 95% confidence interval [CI]: 0.66–1.04, *p* = 0.10) with significant heterogeneity (I^2^ = 81%). Sensitivity analysis of five cohorts of patients with cancer events before the diagnosis of schizophrenia indicated that schizophrenia was associated with a significantly lower incidence of liver cancer (SIR: 0.76, 95% CI: 0.61–0.96, *p* = 0.02; I^2^ = 84%). The reduction of a subsequent incidence of liver cancer was significant in male patients with schizophrenia (SIR: 0.71, *p* = 0.005), and a trend of a reduced risk of liver cancer was also detected in female patients (SIR: 0.83, *p* = 0.12). Significant publication bias was detected. However, “trim and fill” analyses by including the imputed unpublished studies showed similar results. In summary, schizophrenia may be protective against the incidence of liver cancer.

## INTRODUCTION

Schizophrenia is a serious mental disease that exposes the patient to many other clinical disorders, including cancer [1, 2]. Conventionally, patients with schizophrenia are considered to confer a higher risk for cancer incidence because many cancer-related risk factors have been found to be prevalent in these patients, such as smoking [3, 4], alcohol dependence [5, 6], obesity [7, 8], lack of physical exercise [9, 10], and an unhealthy diet [11, 12]. Accordingly, many observational studies have been performed to evaluate the potential risk of cancer incidence and mortality after the diagnosis of schizophrenia [13]. A recently published meta-analysis including [19] studies has shown that patients with schizophrenia have a significantly increased risk of cancer mortality as compared with the general population (pooled standardized mortality ratio: 1.40) [14]. However, regarding the incidence of cancer in patients with schizophrenia, the results of the previous studies are inconsistent [15]. In an early meta-analysis, Catts et al. found that the overall incidence of cancer after the diagnosis of schizophrenia was not statistically different from the general population [16]. The results of subsequent stratified analyses indicated that the associations between schizophrenia and various types of cancer may be different. The incidence rates of lung cancer and breast cancer were found to be significantly higher in patients with schizophrenia as compared with the general population, while the incidence rates of colorectal cancer, prostate cancer, and malignant melanoma were found to be lower [16]. These results suggest the complexity for the association between schizophrenia and cancer incidence. Of note, the potential association between schizophrenia and the risk of liver cancer was not evaluated in the previous meta-analysis due to the limited number of available studies. However, it has been well recognized that the two major risk factors for liver cancer, alcohol consumption 5 and hepatitis B or C [17, 18], are both prevalent in patients with schizophrenia. Moreover, many cohort studies have been reported or updated [19–25] since the last meta-analysis regarding the association between schizophrenia diagnosis and cancer risk. Therefore, in this study, we aimed to perform a systematic review and meta-analysis to evaluate the incidence of liver cancer after the diagnosis of schizophrenia.

## MATERIALS AND METHODS

We followed the Meta-analysis of Observational Studies in Epidemiology (MOOSE) [26] and Cochrane's Handbook [27] guidelines throughout the design, implementation, analysis, and reporting for this study.

### Literature searching

The PubMed and Embase databases were systematically searched for relevant studies with the terms “schizophrenia,” “schizophrenic,” “psychosis,” combined with “liver,” “hepatic,” and “cancer,” “tumor,” “neoplasm,” or “carcinoma.” The search results were limited to studies in humans and published in the English language. We also manually screened the reference lists of the original and review articles. The final literature search was performed on July 14, 2017.

### Inclusion and exclusion criteria

To be included in the current meta-analysis, the studies must have fulfilled the following criteria: 1) articles were full-length and written in English; 2) the studies were designed as cohort studies (prospective or retrospective, regardless of the sample size and follow-up duration); 3) an adult population (≥ 18 years of age) was included; 4) schizophrenia was identified as exposure at baseline; 5) the general population without a diagnosis of schizophrenia was used as a control; 6) the incidence of liver cancer on follow-up was documented; and 7) the adjusted standardized incidence ratios (SIRs, at least adjusted for age and gender) and their corresponding 95% confidence intervals (CIs) for liver cancer incidence in schizophrenic patients as compared with controls were reported. The diagnostic criteria of schizophrenia and the confirmation of liver cancer cases were consistent with those applied in the original articles. If studies with overlapping participants were encountered, the reports with a larger sample size were included in the current meta-analysis. Abstracts, letters to the editor, reviews, and studies with designs other than a cohort study were excluded from the current study. Studies reporting liver cancer-related mortality were also excluded because many factors, such as the comorbidities and treatments between patients with schizophrenia and controls, may further confound the results.

### Data extraction and quality evaluation

Two independent authors performed the literature search, data extraction, and quality assessment, according to the predefined inclusion criteria. Discrepancies were resolved by consensus. Data regarding the publication information (name of first author, year of publication, and country where the study was performed), patient characteristics (numbers, sources of the study populations), follow-up information (years of follow-up, numbers of liver cancer cases), and other characteristics (e.g., whether cases of cancer incidence before the diagnosis of schizophrenia were excluded) were extracted. The Newcastle-Ottawa Scale [28] was used to evaluate the quality of the included studies. This scale ranges from 1 to 9 stars and judges each study regarding the aspects of selection of the study groups, the comparability of the groups, and the ascertainment of the outcome of interest.

### Statistical analyses

Data of SIRs and their corresponding standard errors were calculated from 95% CIs or *p* values, and were logarithmically transformed to stabilize variance and to normalize the distribution. The Cochrane's *Q* test [27] and I^2^ test [29] were used to evaluate the heterogeneity among the included cohort studies. A significant heterogeneity was considered if I^2^ > 50%. A random-effects model was used for the meta-analysis of the SIR data because this model is considered to produce a more generalized result via incorporation of the potential heterogeneity [30]. Subgroup analyses were performed to evaluate whether the gender of the participants significantly affected the association between schizophrenia and liver cancer. Potential publication bias was assessed by funnel plots with the Egger regression asymmetry test [31]. The nonparametric “trim and fill” procedure was also performed to further assess the possible effect of publication bias in our meta-analysis. This method considers the possibility of hypothetical “missing” studies that might exist, imputes their SIRs, and recalculates a pooled SIR that incorporates the hypothetical missing studies as though they actually existed [27]. RevMan (Version 5.1; Cochrane Collaboration, Oxford, UK) and STATA software (Version 12.0; Stata Corporation, College Station, TX) were used for the statistical analyses.

## RESULTS

### Literature search results

**Figure 1:**
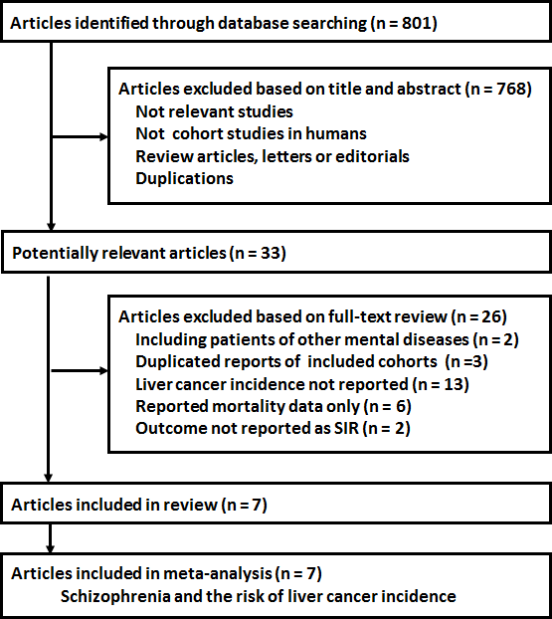
The flowchart of the literature search strategy

The flowchart of the literature search strategy is summarized in Figure 1. Briefly, 801 articles were identified after the initial database search, and 768 articles were excluded after screening via titles and abstracts, mainly because they were irrelevant to the aim of the study. For the [33] studies that underwent full-text review, [26] were excluded mostly because they did not report the outcomes of liver cancer incidence. Finally, seven studies [19–25] were included.

### Study characteristics and quality evaluation

Overall, seven database-based retrospective cohort studies [19–25] were included, with 312,834 patients with schizophrenia. The characteristics of the studies are presented in Table 1. Four of the studies were performed in Europe [19–21, 23], while the other three were in Asia [22, 24, 25]. Five studies included hospitalized patients with schizophrenia [20, 21, 23, 25], while the other two did not specify the source of the patients [22, 24]. The years of follow-up of the included studies varied from 1963 to 2010. A total of 581 cases of liver cancer were recorded during the follow-up period. Five of the studies [20, 22–25] excluded patients with cancer events before the diagnosis of schizophrenia, while the other two did not [19, 21]. Gender-specified SIRs of liver cancer were available from five cohorts [20, 22–25]. The qualities of the included studies were generally good, with a Newcastle-Ottawa Scale score between 6 and 8.

**Table 1:** Characteristics of the included studies

Study	Country	Patient characteristics	Number of patients with schizophrenia	Comparison population	Study years	Confirmation of liver cancer cases	Number of liver cancer cases	SIR outcomes reported	Exclusion of liver cancer incidence before schizophrenia	Quality scores
Lichtermann 2001	Finland	Inpatients or those with disability pension for schizophrenia	26,996	General Finnish population	1971–1996	Finnish Cancer Registry	8	T	NS	7
Goldacre 2005	UK	Inpatients with schizophrenia in Oxford	9,649	General population	1963–1999	National Health Service based data	7	T	NS	6
Dalton 2005	Denmark	Inpatients with schizophrenia	22,766	General Danish population	1969–1995	Danish Cancer Registry	12	M, F	Y	8
Chou 2011	China	Patients diagnosed with schizophrenia in National Health Insurance Research Database	59,257	Age, sex matched individuals	2000–2008	ICD-9 Classification	118	M, F, T	Y	8
Lin 2013	China	Patients diagnosed with schizophrenia	102,202	General population from the health insurance database	1995–2007	National Cancer Database	190	M, F, T	Y	8
Ji 2013	Sweden	Inpatients with schizophrenia	59,233	General Swedish population	1965–2008	National Cancer Registry of Sweden	188	M, F, T	Y	8
Chen 2016	China	Inpatients with schizophrenia	32,731	General population	2000-2010	ICD-9Classification	58	M, F, T	Y	8

SIR, standardized incidence ratio; ICD, international classification of diseases; M, male; F, female; T, total; N, no; Y, yes; NS, not stated.

### Schizophrenia and the incidence of liver cancer in the overall population

**Figure 2:**
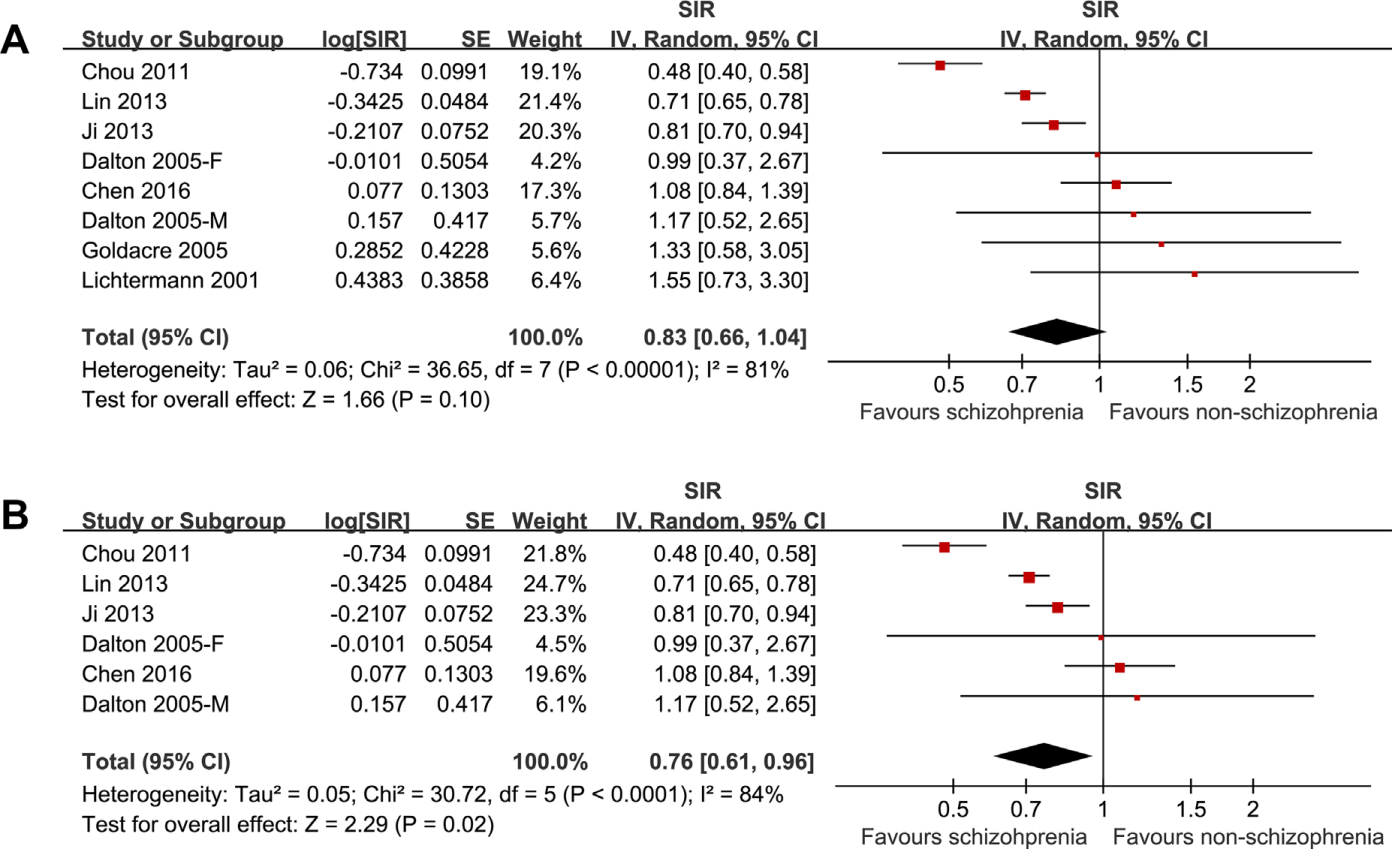
The pooled results of the seven cohorts showed that schizophrenia was associated with a trend of a lower liver cancer incidence with significant heterogeneity

The pooled results of the seven cohorts showed that schizophrenia was associated with a trend of a lower liver cancer incidence (SIR: 0.83, 95% CI: 0.66–1.04, *p* = 0.10; Figure 2A) with significant heterogeneity (*p* for Cochrane's *Q* test < 0.001, I^2^ = 81%). Meta-analysis by pooling the results of five cohorts including patients with cancer events before the diagnosis of schizophrenia showed that schizophrenia was associated with a significantly lower incidence of liver cancer (SIR: 0.76, 95% CI: 0.61–0.96, *p* = 0.02; Figure 2B) with significant heterogeneity (p for Cochrane's *Q* test < 0.001, I^2^ = 84%).

### Schizophrenia and the incidence of liver cancer stratified by gender

**Figure 3:**
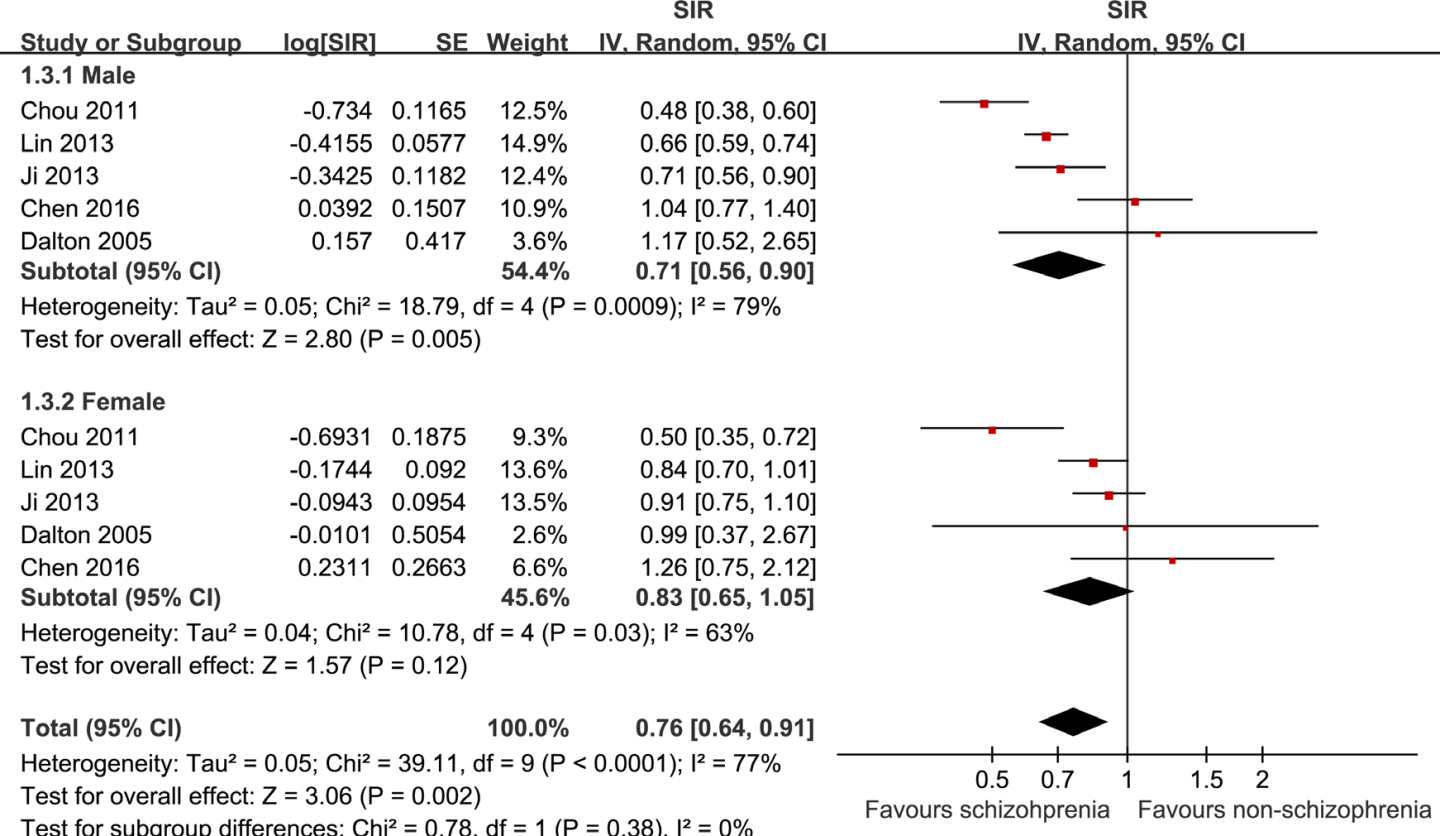
Schizophrenia was associated with a significantly higher incidence of liver cancer in male patients but a nonsignificant trend of a higher incidence of liver cancer in female patients

The pooled results of five cohorts [20, 22–25], which all excluded patients with cancer events before the diagnosis of schizophrenia, showed that schizophrenia was associated with a significantly higher incidence of liver cancer in male patients (SIR: 0.71, 95% CI: 0.56–0.90, *p* = 0.005; I^2^ = 79%; Figure 3), but a nonsignificant trend of a higher incidence of liver cancer in female patients (SIR: 0.83, 95% CI: 0.65–1.05, *p* = 0.12; I^2^ = 63%; Figure 3). The difference between the two subgroups was not statistically significant (*p* = 0.38).

### Publication bias

**Figure 4:**
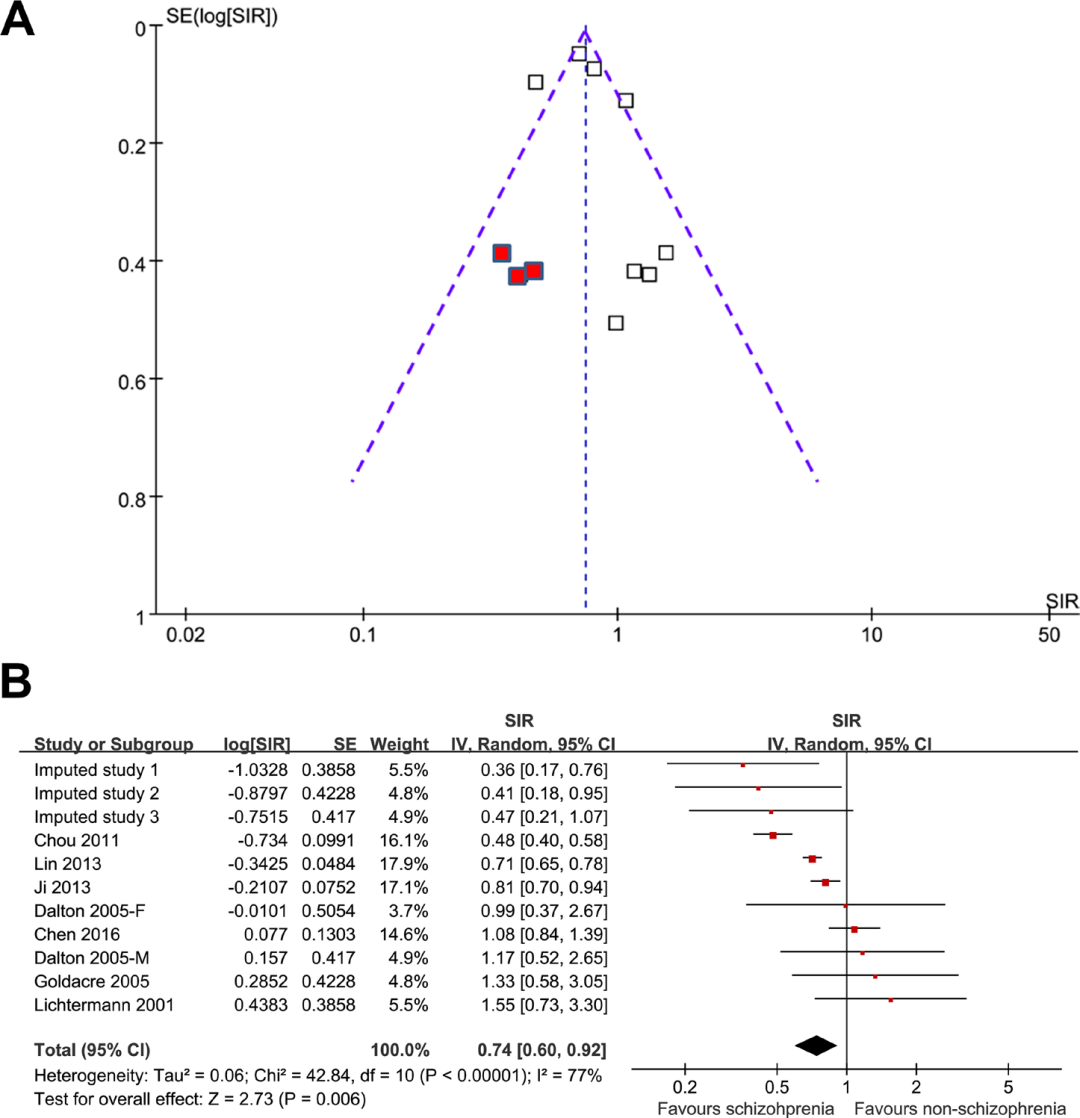
The funnel plot for the association between schizophrenia and lung cancer

The funnel plot for the association between schizophrenia and lung cancer for all participants was asymmetric on visual inspection (Figure 4A), and the results of the Egger regression test also indicated a potential publication bias (*p* = 0.04). After introducing three hypothetical studies by “trim and fill” analysis, the funnel plot became symmetric, and the meta-analysis incorporating the three hypothetical studies showed similar results (SIR: 0.76, 95% CI: 0.64–0.91, *p* = 0.006; I^2^ = 77%; Figure 4B).

## DISCUSSION

In this systematic review and meta-analysis of available cohort studies, we found that patients with schizophrenia were associated with an approximately 20% lowered risk for the incidence of liver cancer as compared with the general population. The reduction of subsequent incidence of liver cancer was significant in male patients with schizophrenia, and a trend of a reduced risk of liver cancer was also detected in female patients. Although the publication bias seems to be significant in this meta-analysis, after inclusion of the imputed unpublished studies with the “trim and fill” method, the pooled results also suggest a significantly lowered incidence of liver cancer in these patients. These findings indicated that, contrary to the conventional supposition, patients with schizophrenia were not associated with a higher risk of liver cancer. Moreover, schizophrenia may be protective against the incidence of liver cancer.

The mechanisms underlying the potential protective effect of schizophrenia against liver cancer are poorly understood. The genetic basis for the potential protective effect of schizophrenia against cancer has been suggested in previous studies showing that the cancer risk in siblings and parents of schizophrenic patients was lower than that of the general population [16]. Genetic polymorphisms of genes involved in the pathogenesis of schizophrenia may also be involved. In a genetic study of schizophrenic Chinese Han patients, XRCC4 gene polymorphisms may be protective against colorectal cancer risk [32]. Similarly, a P53 gene polymorphism was found to be a modifying factor for the susceptibility to lung cancer in patients with schizophrenia 33. Moreover, epigenetic mechanisms also have been suggested to be involved [34, 35]. Studies regarding the genetic association between schizophrenia and liver cancer are rarely reported. However, a previously published genomic study may provide some clues for understanding the association. Using a schizophrenia-hepatocellular carcinoma network, the authors found that some schizophrenia candidate genes (SIRPB1, SYK, and LCK) and genes mediating the immune responses in the etiology mechanism of schizophrenia (IL-2 and TREM-1/DAP12) may also function as tumor-suppressor genes [36]. The results of this study indicate a direct genetic association between schizophrenia and a reduced risk of liver cancer. More studies are needed to elucidate the accurate mechanisms underlying the potential protective effect of schizophrenia against liver cancer.

The results of our study may have important implications for future studies. First, a lower incidence of liver cancer in patients with schizophrenia was confirmed in our study despite the fact that these patients had a higher prevalence of conventional risk factors for liver cancer, such as alcohol consumption and hepatitis B or C. These findings suggest that the high prevalence of the above risk factors may confound the potential association between schizophrenia and liver cancer risk, which should be confirmed in large cohort studies with the adequate adjustment for the alcohol drinking habits and hepatitis B or C prevalence. Moreover, the results of our study highlight the previous paradox of a lower cancer incidence but higher cancer mortality in patients with schizophrenia. In view of the fact that patients with schizophrenia have been found to have poor accessibility to healthcare facilities, these results underline the importance for the improvement of early cancer screening and prevention in these patients [37], which may substantially improve the clinical prognosis of these patients.

Our study has some limitations that should be considered when interpreting the results. First, as mentioned previously, conventional risk factors such as the prevalence of alcohol consumption and the incidence of hepatitis B or C were not adjusted in the included studies when presenting the results. Whether these factors confound the results deserves further investigation. Moreover, many other clinical characteristics of the participants that may also affect the risk of liver cancer, such as the body mass index, occupational information, and dietary habits, were also not controlled in the included studies, which may also confound the association between schizophrenia and liver cancer incidence. In addition, significant heterogeneity was detected, which may be caused by differences of other factors besides the above confounding factors, such as differences of diagnostic criteria for schizophrenia and liver cancer, different medications administered for schizophrenia, and different strategies used to confirm the liver cancer cases. Finally, although we used “trim and fill” analysis to include hypothetical studies, a significant publication bias remained. The results of our meta-analysis should be confirmed in large cohort studies.

## CONCLUSIONS

The current evidence suggests that the diagnosis of schizophrenia at baseline may be protective for the subsequent risk of liver cancer incidence. Future cohort studies with a large sample size and well-controlled confounding factors are needed to confirm our findings.

## References

[1] Chou FH, Tsai KY, Wu HC, Shen SP (2016). Cancer in patients with schizophrenia: What is the next step?. Psychiatry Clin Neurosci.

[2] Howard LM, Barley EA, Davies E, Rigg A, Lempp H, Rose D, Taylor D, Thornicroft G (2010). Cancer diagnosis in people with severe mental illness: practical and ethical issues. Lancet Oncol.

[3] Miyauchi M, Kishida I, Suda A, Shiraishi Y, Fujibayashi M, Taguri M, Ishii C, Ishii N, Moritani T, Hirayasu Y (2017). Long term effects of smoking cessation in hospitalized schizophrenia patients. BMC Psychiatry.

[4] Cather C, Pachas GN, Cieslak KM, Evins AE (2017). Achieving Smoking Cessation in Individuals with Schizophrenia: Special Considerations. CNS Drugs.

[5] Koskinen J, Lohonen J, Koponen H, Isohanni M, Miettunen J (2009). Prevalence of alcohol use disorders in schizophrenia—a systematic review and meta-analysis. Acta Psychiatr Scand.

[6] Chang CK (2016). Impact of additive alcohol and substance use disorders on the mortality of people with schizophrenia and mood disorders. Evid Based Ment Health.

[7] Li Q, Du X, Zhang Y, Yin G, Zhang G, Walss-Bass C, Quevedo J, Soares JC, Xia H, Li X, Zheng Y, Ning Y, Zhang XY (2017). The prevalence, risk factors and clinical correlates of obesity in Chinese patients with schizophrenia. Psychiatry Res.

[8] Sugai T, Suzuki Y, Yamazaki M, Shimoda K, Mori T, Ozeki Y, Matsuda H, Sugawara N, Yasui-Furukori N, Minami Y, Okamoto K, Sagae T, Someya T (2016). High Prevalence of Obesity, Hypertension, Hyperlipidemia, and Diabetes Mellitus in Japanese Outpatients with Schizophrenia: A Nationwide Survey. PLoS One.

[9] Stubbs B, Chen LJ, Chung MS, Ku PW (2017). Physical activity ameliorates the association between sedentary behavior and cardiometabolic risk among inpatients with schizophrenia: A comparison versus controls using accelerometry. Compr Psychiatry.

[10] Vancampfort D, Stubbs B, Probst M, De Hert M, Schuch FB, Mugisha J, Ward PB, Rosenbaum S (2016). Physical activity as a vital sign in patients with schizophrenia: Evidence and clinical recommendations. Schizophr Res.

[11] Gupta A, Craig TK (2009). Diet, smoking and cardiovascular risk in schizophrenia in high and low care supported housing. Epidemiol Psichiatr Soc.

[12] Heald A, Sein K, Anderson S (2015). Diet, exercise and the metabolic syndrome in schizophrenia: A cross-sectional study. Schizophr Res.

[13] Bushe CJ, Hodgson R (2010). Schizophrenia and cancer: in 2010 do we understand the connection?. Can J Psychiatry.

[14] Zhuo C, Tao R, Jiang R, Lin X, Shao M (2017). Cancer mortality in patients with schizophrenia: systematic review and meta-analysis. Br J Psychiatry.

[15] Tabares-Seisdedos R, Dumont N, Baudot A (2011). No paradox, no progress: inverse cancer comorbidity in people with other complex diseases. Lancet Oncol.

[16] Catts VS, Catts SV, O’Toole BI, Frost AD (2008). Cancer incidence in patients with schizophrenia and their first-degree relatives - a meta-analysis. Acta Psychiatr Scand.

[17] Bauer-Staeb C, Jorgensen L, Lewis G, Dalman C, Osborn DP, Hayes JF (2017). Prevalence and risk factors for HIV, hepatitis B, and hepatitis C in people with severe mental illness: a total population study of Sweden. Lancet Psychiatry.

[18] Wang Y, Yu L, Zhou H (2016). Serologic and molecular characteristics of hepatitis B virus infection in vaccinated schizophrenia patients in China. J Infect Dev Ctries.

[19] Lichtermann D, Ekelund J, Pukkala E, Tanskanen A, Lonnqvist J (2001). Incidence of cancer among persons with schizophrenia and their relatives. Arch Gen Psych.

[20] Dalton SO, Mellemkjaer L, Thomassen L, Mortensen PB, Johansen C (2005). Risk for cancer in a cohort of patients hospitalized for schizophrenia in Denmark, 1969–1993. Schizophr Res.

[21] Goldacre MJ, Kurina LM, Wotton CJ, Yeates D, Seagroat V (2005). Schizophrenia and cancer: an epidemiological study. Br J Psychiatry.

[22] Chou FH, Tsai KY, Su CY, Lee CC (2011). The incidence and relative risk factors for developing cancer among patients with schizophrenia: a nine-year follow-up study. Schizophr Res.

[23] Ji J, Sundquist K, Ning Y, Kendler KS, Sundquist J, Chen X (2013). Incidence of cancer in patients with schizophrenia and their first-degree relatives: a population-based study in Sweden. Schizophr Bull.

[24] Lin CY, Lane HY, Chen TT, Wu YH, Wu CY, Wu VY (2013). Inverse association between cancer risks and age in schizophrenic patients: a 12-year nationwide cohort study. Cancer Sci.

[25] Chen LY, Hung YN, Chen YY (2016). Cancer incidence in young and middle-aged people with schizophrenia: nationwide cohort study in Taiwan, 2000-2010. Epidemiol Psychiatr Sci.

[26] Stroup DF, Berlin JA, Morton SC (2000). Meta-analysis of observational studies in epidemiology: a proposal for reporting. Meta-analysis Of Observational Studies in Epidemiology (MOOSE) group JAMA.

[27] Higgins J, Green S (2011). Cochrane Handbook for Systematic Reviews of Interventions Version 5.1.0. The Cochrane Collaboration.

[28] Wells GA, Shea B, O’Connell D (2010). The Newcastle-Ottawa Scale (NOS) for assessing the quality of nonrandomised studies in meta-analyses. http://www.ohri.ca/programs/clinical_epidemiology/oxford.asp.

[29] Higgins JP, Thompson SG (2002). Quantifying heterogeneity in a meta-analysis. Stat Med.

[30] Patsopoulos NA, Evangelou E, Ioannidis JP (2008). Sensitivity of between-study heterogeneity in meta-analysis: proposed metrics and empirical evaluation. Int J Epidemiol.

[31] Egger M, Davey Smith G, Schneider M, Minder C (1997). Bias in meta-analysis detected by a simple, graphical test. BMJ.

[32] Wang Y, Wang L, Li X (2010). Polymorphisms of XRCC4 are involved in reduced colorectal cancer risk in Chinese schizophrenia patients. BMC Cancer.

[33] Ozbey U, Yuce H, Namli M, Elkiran T (2011). Investigation of Differences in P53 Gene Polymorphisms between Schizophrenia and Lung Cancer Patients in the Turkish Population. Genet Res Int.

[34] Rizos E, Siafakas N, Skourti E, Papageorgiou C, Tsoporis J, Parker TH, Christodoulou DI, Spandidos DA, Katsantoni E, Zoumpourlis V (2016). miRNAs and their role in the correlation between schizophrenia and cancer (Review). Mol Med Rep.

[35] Cromby J, Chung E, Papadopoulos D, Talbot C (2016). Reviewing the epigenetics of schizophrenia. J Ment Health.

[36] Huang KC, Yang KC, Lin H, Tsao Tsun-Hui T, Lee WK, Lee SA, Kao CY (2013). Analysis of schizophrenia and hepatocellular carcinoma genetic network with corresponding modularity and pathways: novel insights to the immune system. BMC Genomics.

[37] Irwin KE, Henderson DC, Knight HP, Pirl WF (2014). Cancer care for individuals with schizophrenia. Cancer.

